# Survey data on the attitudes towards digital technologies and the way of managing e-governmental tasks

**DOI:** 10.1016/j.dib.2022.108871

**Published:** 2022-12-31

**Authors:** Tamás Kaiser, László Gadár

**Affiliations:** a University of Public Service, Budapest, Hungary; b ELKH-PE Complex Systems Monitoring Research Group, University of Pannonia, Veszprém, Hungary

**Keywords:** Online questionnaire, Representative sample, Digital competencies, Characteristics of technophiles, Smart device usage, Purpose of internet usage, E-government, public administration

## Abstract

A representative sample of 2520 people was surveyed by completing an online questionnaire using the Tablet Assisted Personal Interviewing (TAPI) method to measure the digital readiness and administrative habits of the population. The data are available in tabular form from an open repository. Some topics were processed by measuring several latent variables and latent class analysis was applied. The database facilitated understanding of the stratification of the population according to digital literacy, preferences with regard to device usage, the purpose of Internet usage, fear of smart systems as well as technophilic and technophobic attitudes. The database revealed the population's administrative practices, particularly their attitudes towards e-government and future development needs. The survey is useful for planning e-government developments. Knowledge of the digital readiness of the population is useful for designing training programs, mapping labor market competencies in the era of Industry 4.0 and developing channels of communication campaigning. Through the regional demographic variables, all of the aforementioned topics can also be used in regional science to measure indices at NUTS 2 or NUTS 3 levels.

**Specifications Table**

**Table:** 

Subject	Social science
Specific subject area	digital technology-related behavior, technology-driven administration, paperless administration, definition of target groups
Type of data	Table in .csv and data labels in .txt format.
How the data were acquired	Survey data were gathered using the LimeSurvey survey tool hosted on our server.
Data format	Raw, processed
Description of data collection	The survey was answered by 2520 respondents by following the TAPI (Tablet Assisted Personal Interviewing) method. This sample of Hungarian society is representative of gender, age, place of residence and level of education among the population over the age of 18. 1259 individuals from the sample, which is representative in itself, were asked a series of questions related to smart applications and the use of smart devices. Another 1261 individuals from the sample were asked about the importance of some digital developments related to public administration. The sample was split to reduce the burden on respondents. The addresses of those involved in the research were selected by multistage, proportionally stratified probability sampling. Data were collected between 7 March and 15 April 2020 in person from the address of the respondents. Regarding the COVID-19 pandemic, the company conducting the survey provided the opportunity to respond by telephone following a personal inquiry, which was taken up by 76% of the respondents.
Data source location	Country: Hungary City/Region: all over regions of Hungary
Description of data location	The data originated from University of Public Service, Budapest, Hungary.
Data accessibility	Demeter, Endre; Petényi, Sára; Kaiser, Tamás; Gadár, László (2022): “Survey data on the attitudes towards digital technologies and the methods of doing citizens’ (governmental) administration tasks”, Mendeley Data, V3, doi: 10.17632/typp3nymmr.3 https://data.mendeley.com/datasets/typp3nymmr/3

## Value of the Data

• The database is representative of society in terms of gender, age, type of settlement and level of education.
• It covers a wide range of topics to help understand the spread of digitalization in terms of using smart devices and applications, especially in administration as well as shows the interest in technological openness, Internet usage habits and behavior, e-government developments and demographic characteristics and provides novel insights into the background of digital devices and applications used in terms of e-government developments.
• The information extracted from the database can fine-tune the objectives and performance evaluation of future digital development projects.
• The database is useful for regional researchers, public administration experts, back offices of ministries, researchers, agencies, think tanks and consultancies to get to know their clients better. as well as smart device and application developers.
• The background behind international and regional indicators (e.g. from the World Bank, OECD) can be identified and explanatory factors found by taking regional data into account because NUTS 2 and 3 codes of residence are included in the database.

## 1 Objective

The rise of digital solutions and devices is bringing a lot of changes and challenges to our everyday lives. Citizens react to these changes in different ways. Public administration is also going digital and should not exclude any citizen. The purpose of this survey was to compile a multi-faceted measurement tool that can capture societal responses to digitization in the context of eGovernment developments. The database will add value to much research on the spread of digitalization knowledge, preparing eGovernment developments, and determining the target and excluded groups to understand their background better.

## 2 Data Description

**Fig. 1:**
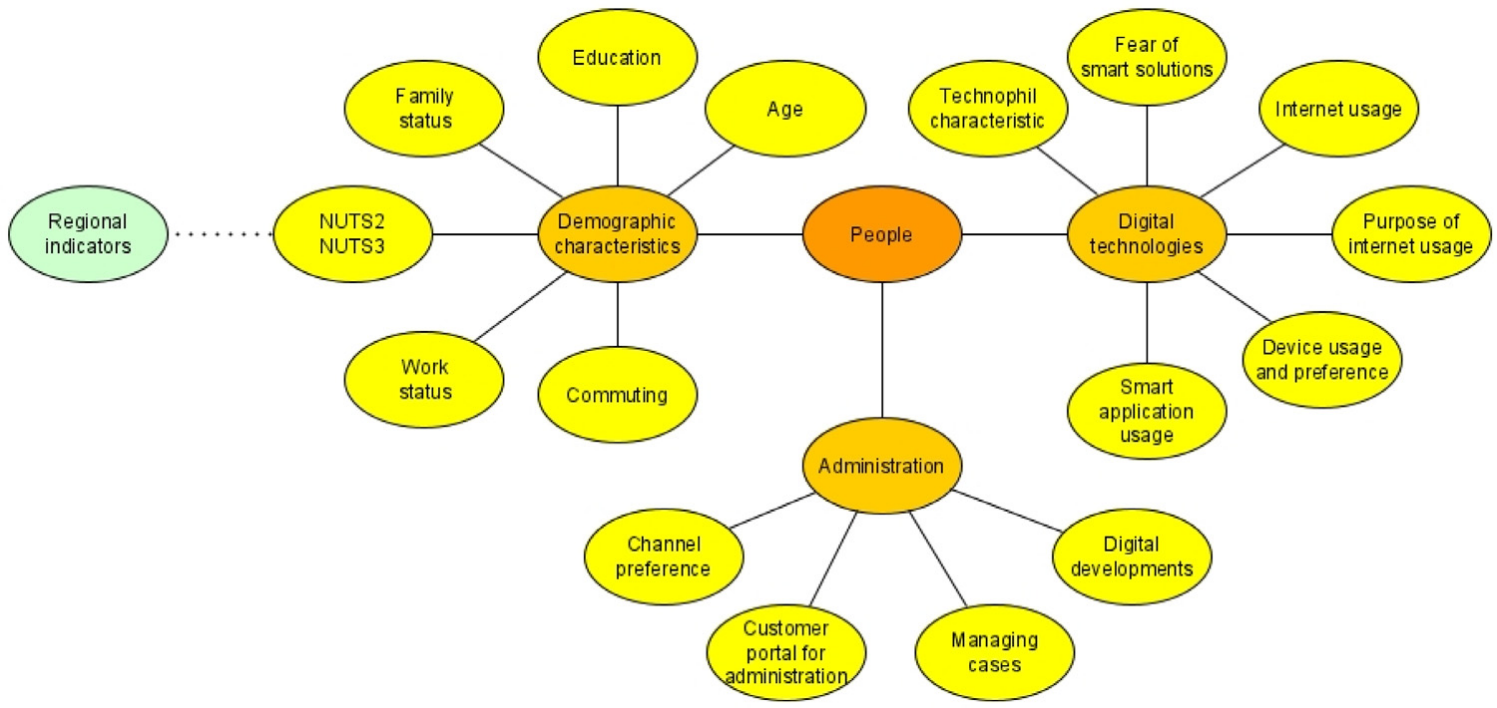
Subject areas covered by the database.

Systematically surveying stakeholders can assess attitudes towards digital developments and technological systems. The topics investigated are presented in Fig. 1. The measures of each factor help to identify novel correlations. The database can be used to formulate several research questions and answers.

2520 people participated in our survey. The respondents are representative of the population of Hungary in terms of age, gender, type of settlement and level of education. Respondents were contacted by telephone concerning the COVID-19 pandemic. The questionnaires were completed using the TAPI method. Although a Likert scale was used for the majority of questions, categorical responses were requested in several cases and in some cases multiple choice questions were appropriate when measuring certain topics. In the following three subsections, the topics assessed are described and the distributions of the response categories given.

The measured variables can be grouped as follows:

• Demographic variables
  ○ Region and subregion (D1, D2)
  ○ Classification of settlement (D3, D12)
  ○ Gender (D4)
  ○ Age (D5)
  ○ Level of education (D6)
  ○ Family status (D7)
  ○ Size of household, number of children under 18 years old (D8, D9)
  ○ Employment status, income level (D10, D11)
  ○ Commuting characteristics of rural people (D13-D20)
• Attitudes towards digital technologies
  ○ Technophile - technophobe (part 1 of sample) (T1-T5)
  ○ Fear of digital technologies (part 1 of sample) (T6-T15)
  ○ Internet usage (T16-T19)
  ○ Purpose of Internet usage (who use the Internet at least every two weeks, T17=1) (T20-T33)
  ○ Preferred and used device when using the Internet (who use the Internet at least every two weeks) (T34-T39)
  ○ Use of smart applications (part 1 of sample) (T40-T51)
• Administration
  ○ Preferred channel for administration (who use the Internet at least every two weeks) (A1-A4), avoid the use of any channels (A5-A8), and preference for a particular channel (A9)
  ○ Having (A10) and using (A11) at least one phone number or web page (A12-A13) for public administration
  ○ Usage of a customer portal for public administration (A14-A16)
  ○ Aspects considered important for maintaining administrative efficiency, channel (A17-A32) and rank (A33-A48) (part 2 of sample)
  ○ Openness to trying out some improvements (A49-A52)
  ○ Demands for some degree of development in administration (A53-A65)

The questionnaire was split so part 1 of the sample were asked some questions, while part 2 were asked others to reduce the burden on respondents. Identifying correlations between these question blocks was challenging.

The survey data were supplemented with clustered variables (D19, D20, T4, T33, A4, A65), which was beneficial to reduce the information content and smooth out the variance in responses by considering some similar variables. An efficient and powerful statistical method was applied for clustering, namely Latent Class Analysis (LCA), which has been successfully used in other fields of social science research given its many advantages. The technique measures a so-called latent variable by taking categorical indicators into account and classifies each set of measurements, in our case, the respondents, into classes according to the latent variable. LCA creates mutually exclusive latent classes, i.e. groups of individuals, based on similar response patterns to the indicator variables.

### 2.1 Demographic data

The names of the variables describing demography in the database start with 'D' denoting the word ‘demography’. The variation in the demographic characteristics of respondents by question is shown below.

*D1: County of residence (NUTS 3)*

**Table:** 

Answers	Number of respondents	Proportion of respondents (%)
1: HU331 (Bács-Kiskun) 2: HU231 (Baranya) 3: HU332 (Békés) 4: HU331 (Borsod-Abaúj-Zemplén) 5: HU333 (Csongrád) 6: HU221 (Fejér) 7: HU221 (Győr-Moson-Sopron) 8: HU321 (Hajdú-Bihar)	134 93 90 166 112 108 103 134	5.3 3.7 3.6 6.6 4.4 4.3 4.1 5.3
9: HU312 (Heves) 10: HU322 (Jász-Nagykun-Szolnok) 11: HU212 (Komárom-Esztergom) 12: HU313 (Nógrád) 13: HU120 (Pest, excluding the capital city of Budapest) 14: HU232 (Somogy) 15: HU323 (Szabolcs-Szatmár-Bereg) 16: HU233 (Tolna) 17: HU222 (Vas) 18: HU213 (Veszprém) 19: HU223 (Zala) 20: part of HU120 - the capital city of Budapest	84 96 77 44 323 93 139 51 67 93 74 439	3.3 3.8 3.1 1.7 12.8 3.7 5.5 2.0 2.7 3.7 2.9 17.4

*D2: Region of residence (NUTS 2)*

**Table:** 

Answers	Number of respondents	Proportion of respondents (%)
1: HU11 (Budapest) 2: HU31 (Észak-Magyarország) 3: HU32 (Észak-Alföld) 4: HU33 (Dél-Alföld) 5: HU12 (Pest megye) 6: HU22 (Nyugat-Dunántúl) 7: HU21 (Közép-Dunántúl) 8: HU23 (Dél-Dunántúl)	439 294 369 336 323 244 278 237	17.4 11.7 14.6 13.3 12.8 9.7 11.0 9.4

*D3: Administrative classification of the settlement*

**Table:** 

Answers	Number of respondents	Proportion of respondents (%)
1: The capital city of Budapest 2: County seat 3: Other city 4: Village	439 523 809 749	17.4 20.8 32.1 29.7

*D4: Gender*

**Table:** 

Answers	Number of respondents	Proportion of respondents (%)
1: Male 2: Female	1184 1336	47.0 53.0

*D5: Age*

**Table:** 

Answers	Number of respondents	Proportion of respondents (%)
1: 18–29 years old 2: 30–39 years old 3: 40–49 years old 4: 50–59 years old 5: 60–69 years old 6: 70+ years old	424 404 475 395 423 399	16.8 16.0 18.8 15.7 16.8 15.8

*D6: Highest Level of Education*

**Table:** 

Answers	Number of respondents	Proportion of respondents (%)
1: Primary or lower school 2: Vocational school 3: Secondary school 4: Higher education or higher	854 522 798 346	33.9 20.7 31.7 13.7

*D7: Family status*

**Table:** 

Answers	Number of respondents	Proportion of respondents (%)
1: Single 2: Cohabiting 3: Married 4: Divorced 5: Widower 88: Do not know 99: No answer	437 419 1171 253 237 2 1	17.4 16.6 46.5 10.1 9.4 - -

*D8: How many people live in your household?*

**Table:** 

Answers	Number of respondents	Proportion of respondents (%)
1: 1 person 2: 2 people 3: 3 people 4: 4 people 5: 5 people 6: 6 people or more	443 994 550 378 110 45	17.6 39.4 21.8 15.0 4.4 1.8

*D9: How many in your household are children under 18?*

**Table:** 

Answers	Number of respondents	Proportion of respondents (%)
0: 0 people 1: 1 person 2: 2 people 3: 3 people or more 99: No answer	1420 374 224 59 443	68.4 18.0 10.8 2.8 -

*D10: Which employment status is most descriptive of you?*

**Table:** 

Answers	Number of respondents	Proportion of respondents (%)
1: Full-time worker 2: Part-time worker 3: Entrepreneur 4: Public worker (financed by the government) 5: Unemployed 6: Full-time mother, home care worker 7: On maternity leave at home 8: Pensioner 9: Disability pensioner 10: Student 11: Other	1317 50 113 8 62 24 52 689 88 107 10	52.3 2.0 4.5 0.3 2.5 1.0 2.1 27.3 3.5 4.2 0.4

*D11: Level of Income*

**Table:** 

Answers	Number of respondents	Proportion of respondents (%)
1: 0 – 150,000 HUF (considered poor) 2: 150,001 – 300,000 HUF (below-average) 3: 300,001 – 500,000 HUF (above-average) 4: 500,001 – 1000,000 HUF (considered to be a high standard of living) 5: 1000,001+ HUF (considered rich) 99: No answer	303 803 678 159 12 565	15.5 41.1 34.7 8.1 0.6 -

*D12: Please classify your place of residence.*

**Table:** 

Answers	Number of respondents	Proportion of respondents (%)
1: Farm 2: Village 3: Low-income part of the municipality (e.g. in gipsy colony) 4: Suburb with detached houses 5: Suburb with apartment buildings 6: Housing estate 7: City center	17 680 14 889 243 485 192	0.7 27.0 0.6 35.3 9.6 19.2 7.6

*D13: How often do you travel to the county seat?* (respondents not living in a county seat or the capital city, D3=3 or D3=4) 780 people continuous variable (0–35)

99: No answer

*D14: How often do you travel to the center of the subregion?* (respondents not living in a county seat or the capital city, D3=3 or D3=4) 780 people continuous variable (0–35)

99: No answer

*D15: How often do you travel to the nearest city?* (respondents not living in a county seat or the capital city, D3=3 or D3=4) 780 people continuous variable (0–35)

99: No answer

*D16: How often do you travel to the county seat?* (categorical version of D13)

**Table:** 

Answers	Number of respondents	Proportion of respondents (%)
1: 0 (never) 2: 1–5 (once a week) 3: 6–14 (several times a week) 4: 15–35 (often, almost every day or every weekday, commuters) 99: No answer	235 372 73 85 1755	30.7 48.6 9.5 11.1 -

*D17: How often do you travel to the center of the subregion?* (categorical version of D14)

**Table:** 

Answers	Number of respondents	Proportion of respondents (%)
1: 0 (never) 2: 1–5 (once a week) 3: 6–14 (several times a week) 4: 15–35 (often, almost every day or every weekday, commuters) 99: No answer	300 335 51 64 1770	40.0 44.7 6.8 8.5 -

*D18: How often do you travel to the nearest city?* (categorical version of D15)

**Table:** 

Answers	Number of respondents	Proportion of respondents (%)
1: 0 (never) 2: 1–5 (once a week) 3: 6–14 (several times a week) 4: 15–35 (often, almost every day or every weekday, commuters) 99: No answer	143 411 91 108 1767	19.0 54.6 12.1 14.3 -

*D19: Summary variable with regard to the intensity of commuting of rural residents, clustered by LCA using variables D16–18.* (for further details, see below)

**Table:** 

Answers	Number of respondents	Proportion of respondents (%)
1: Never commutes 2: Rarely commutes 3: Rarely commutes to the county seat 4: Rarely commutes to the nearest city 5: Medium level of commuting 6: Frequently commutes to the nearest city 7: Frequently commutes to the county seat 8: Very frequently commutes 99: No answer	69 65 130 294 55 44 40 83 1740	8.8 8.3 16.7 37.7 7.1 5.6 5.12 10.6 -

*D20: Summary variable with regard to the intensity of commuting of rural residents, clustered by LCA using variables D16–18.*

**Table:** 

Answers	Number of respondents	Proportion of respondents (%)
1: Never commutes 2: Rarely commutes 3: Frequently commutes 99: No answer	69 544 167 1740	8.8 69.7 21.4 -

**Fig. 2:**
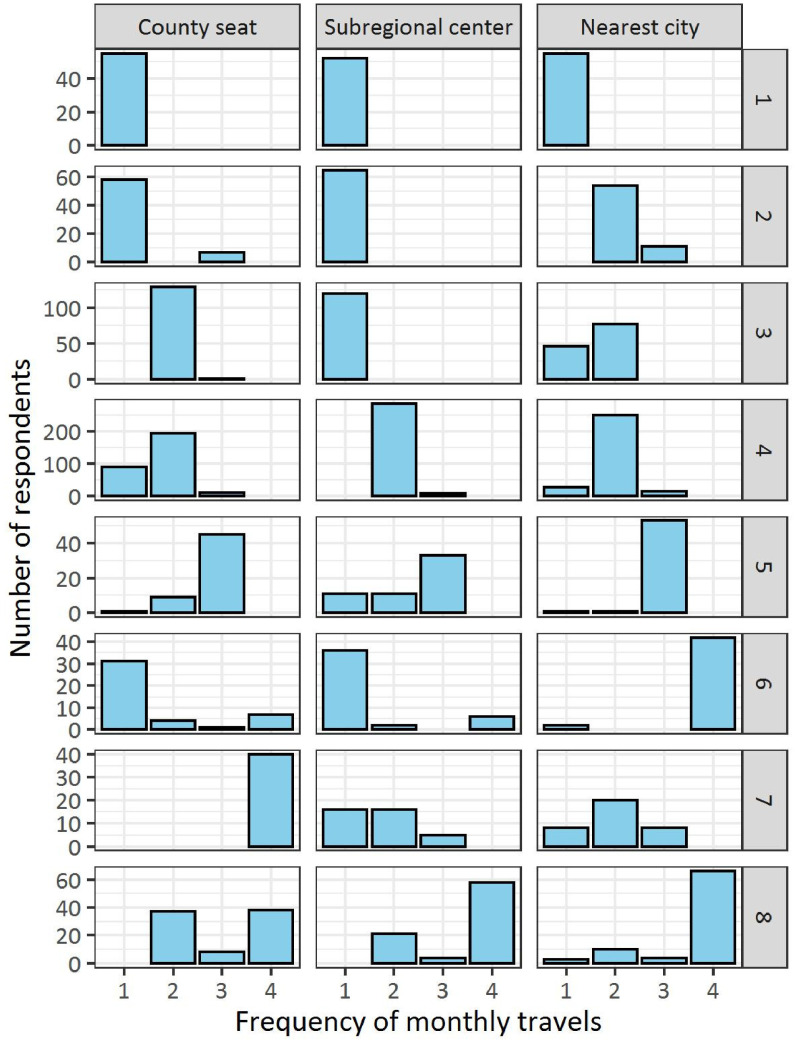
Distribution of the responses of respondents in different commuting clusters (rows denote groups of respondents exhibiting different commuting behaviors, while columns represent the city type of the destination).

Variable D19 is clustered considering the variables D16–18. Our aim was to determine the frequency of commuting and destination of rural residents to summarize as well as simplify related continuous variables. In our case, the indicator variable was the frequency of travel to a given destination (D16–18) and the latent variable was commuting (D19). The calculations were performed using LCA in R with poLCA [1] package. The distribution of the responses of respondents in each group is shown in Fig. 2. The categories of variable D19 were named based on Fig. 2.

### 2.2 Digital technologies

The names of the variables describing demography in the database start with 'T', denoting the word ‘technology’. The distribution of respondents' attitudes towards and characteristics with regard to digital technologies by question are shown below:

*T1: I like trying new technological tools.*

**Table:** 

Answers	Number of respondents	Proportion of respondents (%)
1: Strongly disagree 2: Disagree 3: Agree 4: Strongly agree 88: Do not know 99: No answer	212 282 460 292 13 1261	17.0 22.6 36.9 23.4 - -

*T2: I am afraid of using technologies that I am not familiar with.*

**Table:** 

Answers	Number of respondents	Proportion of respondents (%)
1: Strongly disagree 2: Disagree 3: Agree 4: Strongly agree 88: Do not know 99: No answer	220 389 342 294 14 1261	17.7 31.2 27.5 23.6 - -

*T3: Usually the amount of energy required to learn about technological innovations outweighs their benefits.*

**Table:** 

Answers	Number of respondents	Proportion of respondents (%)
1: Strongly disagree 2: Disagree 3: Agree 4: Strongly agree 88: Do not know 99: No answer	184 405 386 207 77 1261	15.6 34.3 32.7 17.5 - -

*T4: I don't want to miss out on new smart solutions.*

**Table:** 

Answers	Number of respondents	Proportion of respondents (%)
1: Strongly disagree 2: Disagree 3: Agree 4: Strongly agree 88: Do not know 99: No answer	199 340 505 182 33 1261	16.2 27.7 41.2 14.8 - -

*T5: Summary variable of technological openness (technophilic or technophobic behavior), clustered by LCA using variables T1–4.*

**Table:** 

Answers	Number of respondents	Proportion of respondents (%)
1: Laggards 2: Late adopters 3: Early adopters 4: Innovators 99: No answer	213 256 450 246 1355	18.3 22.0 38.6 21.1 -

**Fig. 3:**
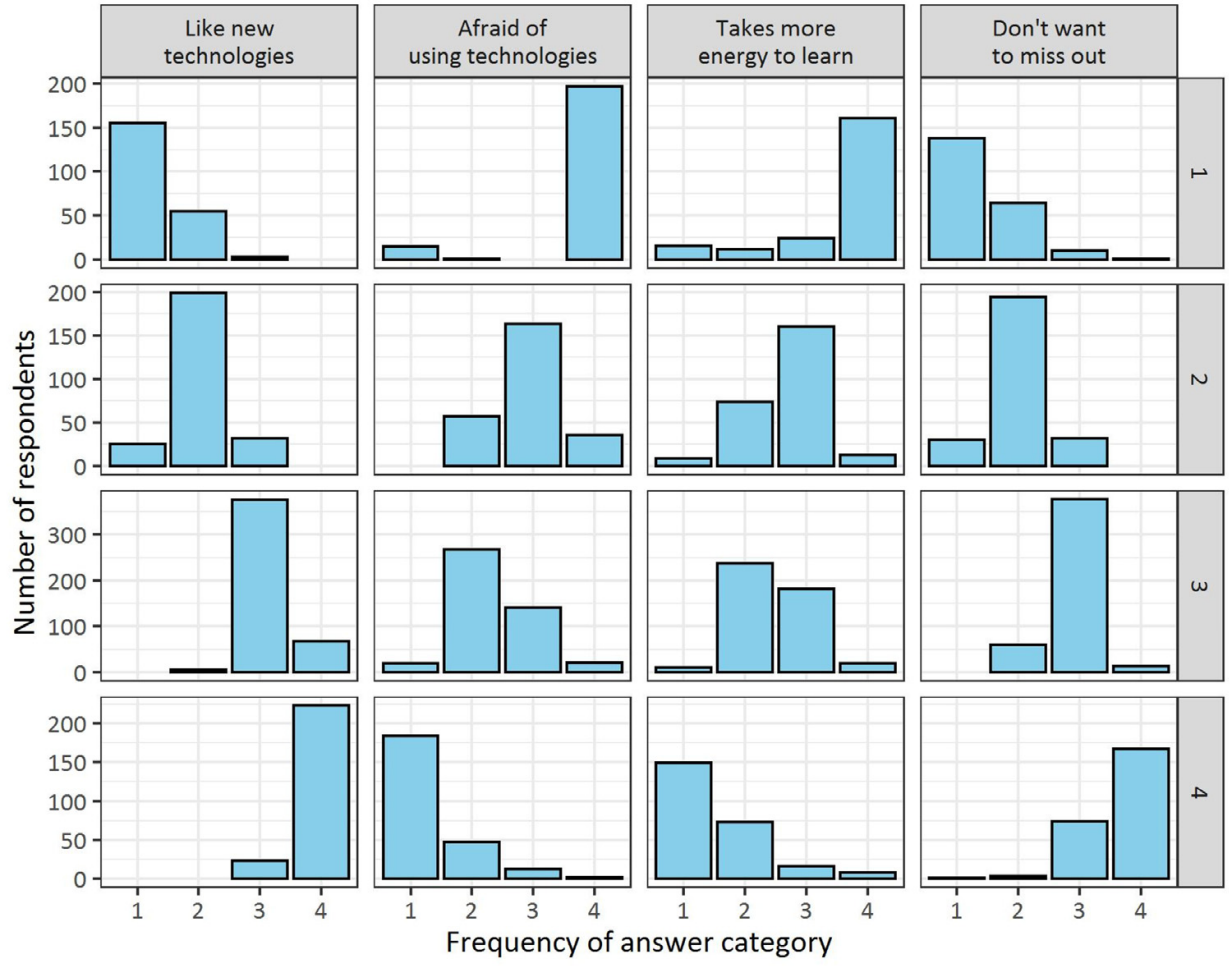
Distributions of the answers of respondents exhibiting different levels of openness to technologies (rows denote groups of respondents, while columns represent variables related to technological openness: T1–4).

Variable T5 is the result of variables T1–4 and is a summary of them. Variable T5 is constructed in the same way as D19, using LCA, which summarizes the respondents' openness to technology, that is, their technophilic or technophobic behavior. The distribution of responses with regard to the respondent clusters is shown in Fig. 3, which can be used to distinguish between the Rogersian-like categories [2] of innovators, early adopters, late adopters and laggards.

*T6: Which of the following factors prevent you from using smart solutions: accessibility/availability*

**Table:** 

Answers	Number of respondents	Proportion of respondents (%)
0: No 1: Yes 99: No answer	1003 256 1261	79.7 20.3 -

*T7: Which of the following factors prevent you from using smart solutions: difficult to use*

**Table:** 

Answers	Number of respondents	Proportion of respondents (%)
0: No 1: Yes 99: No answer	744 515 1261	59.1 40.9 -

*T8: Which of the following factors prevent you from using smart solutions: no native language menu*

**Table:** 

Answers	Number of respondents	Proportion of respondents (%)
0: No 1: Yes 99: No answer	1014 245 1261	80.5 19.5 -

*T9: Which of the following factors prevent you from using smart solutions: data security not guaranteed*

**Table:** 

Answers	Number of respondents	Proportion of respondents (%)
0: No 1: Yes 99: No answer	1088 171 1261	86.4 13.6 -

*T10: Which of the following factors prevent you from using smart solutions: personal fears (*e.g. *making a mistake)*

**Table:** 

Answers	Number of respondents	Proportion of respondents (%)
0: No 1: Yes 99: No answer	1060 199 1261	84.2 15.8 -

*T11: Which of the following factors prevent you from using smart solutions: unreliability of the manufacturer*

**Table:** 

Answers	Number of respondents	Proportion of respondents (%)
0: No 1: Yes 99: No answer	1063 196 1261	84.4 15.6 -

*T12: Which of the following factors prevent you from using smart solutions: discontinuous operation*

**Table:** 

Answers	Number of respondents	Proportion of respondents (%)
0: No 1: Yes 99: No answer	997 262 1261	79.2 20.8 -

*T13: Which of the following factors prevent you from using smart solutions: aversion to smart solutions*

**Table:** 

Answers	Number of respondents	Proportion of respondents (%)
0: No 1: Yes 99: No answer	1092 167 1261	86.7 13.3 -

*T14: Which of the following factors prevent you from using smart solutions: lack of willpower*

**Table:** 

Answers	Number of respondents	Proportion of respondents (%)
0: No 1: Yes 99: No answer	1102 157 1261	87.6 12.5 -

*T15: Which of the following factors prevent you from using smart solutions: other*

**Table:** 

Answers	Number of respondents	Proportion of respondents (%)
0: No 1: Yes 99: No answer	1091 168 1261	86.7 13.3 -

*T16: How often do you use the Internet?*

**Table:** 

Answers	Number of respondents	Proportion of respondents (%)
1: Never 2: Not used for a year 3: Once or twice in the last six months 4: Once a month 5: Once every two weeks 6: Once a week 7: Two-three times a week 8: Four-five times a week 9: Every day 10: Continuously online	496 12 4 8 7 31 102 192 1094 574	19.7 0.5 0.2 0.3 0.3 1.2 4.0 7.6 43.4 22.8

*T17: Internet usageat least once every two weeks. (dummy variable derived from variable T6: 1 if answer is equal to 5–10, 0 otherwise).*

**Table:** 

Answers	Number of respondents	Proportion of respondents (%)
0: No 1: Yes	520 2000	20.6 79.4

*T18: Is your household connected to the Internet (wired or wireless)?*

**Table:** 

Answers	Number of respondents	Proportion of respondents (%)
0: No 1: Yes 2: Type of connection is unknown	637 1875 8	25.3 74.4 0.3

*T19: Do you use Mobile Internet?*

**Table:** 

Answers	Number of respondents	Proportion of respondents (%)
0: No 1: Yes 2: Type of connection is unknown	1006 1503 11	39.9 59.6 0.4

*T20: How often do you do the following activities online: use search engines*

**Table:** 

Answers	Number of respondents	Proportion of respondents (%)
1: Never 2: Rarely 3: Once or twice a week 4: Every day, almost every day 99: No answer	27 227 695 1051 520	1.4 11.3 34.8 52.5 -

*T21: How often do you do the following activities online: view news portals*

**Table:** 

Answers	Number of respondents	Proportion of respondents (%)
1: Never 2: Rarely 3: Once or twice a week 4: Every day, almost every day 99: No answer	86 262 652 1000 520	4.3 13.1 32.6 50.0 -

*T22: How often do you do the following activities online: send and receive emails*

**Table:** 

Answers	Number of respondents	Proportion of respondents (%)
1: Never 2: Rarely 3: Once or twice a week 4: Every day, almost every day 99: No answer	181 645 634 540 520	9.1 32.2 31.7 27.0 -

*T23: How often do you do the following activities online: instant message,* e.g. *Viber, Messenger, etc.*

**Table:** 

Answers	Number of respondents	Proportion of respondents (%)
1: Never 2: Rarely 3: Once or twice a week 4: Every day, almost every day 88: Do not know 99: No answer	176 251 506 1066 1 520	8.8 12.6 25.3 53.3 - -

*T24: How often do you do the following activities online: use social networks,* e.g. *Facebook, Twitter, etc.*

**Table:** 

Answers	Number of respondents	Proportion of respondents (%)
1: Never 2: Rarely 3: Once or twice a week 4: Every day, almost every day 88: Do not know 99: No answer	164 123 404 1308 1 520	8.2 6.2 20.2 65.4 - -

*T25: How often do you do the following activities online: make online calls,* e.g. *on Skype*

**Table:** 

Answers	Number of respondents	Proportion of respondents (%)
1: Never 2: Rarely 3: Once or twice a week 4: Every day, almost every day 88: Do not know 99: No answer	508 558 620 313 1 520	25.4 27.9 31.0 15.7 - -

*T26: How often do you do the following activities online: do online training courses*

**Table:** 

Answers	Number of respondents	Proportion of respondents (%)
1: Never 2: Rarely 3: Once or twice a week 4: Every day, almost every day 88: Do not know 99: No answer	1339 326 201 131 3 520	67.1 16.3 10.1 6.5 - -

*T27: How often do you do the following activities online: work*

**Table:** 

Answers	Number of respondents	Proportion of respondents (%)
1: Never 2: Rarely 3: Once or twice a week 4: Every day, almost every day 88: Do not know 99: No answer	1112 265 242 367 14 520	56.0 13.3 12.2 18.5 - -

*T28: How often do you do the following activities online: shop online*

**Table:** 

Answers	Number of respondents	Proportion of respondents (%)
1: Never 2: Rarely 3: Once or twice a week 4: Every day, almost every day 88: Do not know 99: No answer	630 1171 177 20 2 520	31.5 58.6 8.9 1.0 - -

*T29: How often do you do the following activities online: trade online*

**Table:** 

Answers	Number of respondents	Proportion of respondents (%)
1: Never 2: Rarely 3: Once or twice a week 4: Every day, almost every day 88: Do not know 99: No answer	1283 604 88 22 3 520	64.2 30.2 4.4 1.1 - -

*T30: How often do you do the following activities online: online banking*

**Table:** 

Answers	Number of respondents	Proportion of respondents (%)
1: Never 2: Rarely 3: Once or twice a week 4: Every day, almost every day 88: Do not know 99: No answer	946 795 228 29 2 520	47.3 39.8 11.4 1.5 - -

*T31: How often do you do the following activities online: pay utility bills online*

**Table:** 

Answers	Number of respondents	Proportion of respondents (%)
1: Never 2: Rarely 3: Once or twice a week 4: Every day, almost every day 88: Do not know 99: No answer	1183 655 139 17 5 521	59.3 32.8 7.0 0.9 - -

*T32: How often do you do the following activities online: online administration*

**Table:** 

Answers	Number of respondents	Proportion of respondents (%)
1: Never 2: Rarely 3: Once or twice a week 4: Every day, almost every day 88: Do not know 99: No answer	1150 712 103 23 12 520	57.8 35.8 5.2 1.2 - -

*T33: Summary variable with regard to the purpose of Internet usage clustered by LCA using variables T8–20.*

**Table:** 

Answers	Number of respondents	Proportion of respondents (%)
1: Rarely used 2: Consumers 3: Consumers who shop 4: Consumers who shop and do administration 5: Very rarely productive 6: Rarely productive 7: Productive 8: Very productive 99: No answer	362 239 456 279 153 58 322 103 548	18.4 12.1 23.1 14.1 7.8 2.9 16.3 5.2 -

Variable T33 is the result of variables *T8–20* and is a summary of them. Variable T33 is constructed in the same way as T5 and D19 by applying LCA. Variable T33 separates respondents whose habits with regard to Internet usage are different. For different reasons, everyone uses the Internet for different purposes to satisfy various personal needs in their everyday lives. Consumers are referred to as those who only use search engines, read news stories as well as send and receive emails. Productive respondents work, shop, bank and carry out administrative tasks online on at least a weekly basis. This database offers an opportunity to explore causal effects in terms of behavior and can be used to analyze the consequences of different purposes of Internet usage.

*T34: Do you use a smartphone?*

**Table:** 

Answers	Number of respondents	Proportion of respondents (%)
0: No 1: Yes 2: Do not know what a smartphone is	728 1785 7	28.9 70.8 0.3

*T35: Do you use a smartwatch?*

**Table:** 

Answers	Number of respondents	Proportion of respondents (%)
0: No 1: Yes 2: Do not know what a smartwatch is	2272 212 36	90.2 8.4 1.4

*T36: Which device do you use to access the Internet: a desktop computer or a laptop*

**Table:** 

Answers	Number of respondents	Proportion of respondents (%)
0: No 1: Yes 99: No answer	471 1529 520	23.6 76.4 -

*T37: Which device do you use to access the Internet: a smartphone or a tablet*

**Table:** 

Answers	Number of respondents	Proportion of respondents (%)
0: No 1: Yes 99: No answer	330 1670 520	16.5 83.5 -

*T38: I prefer to do tasks on a desktop computer or laptop rather than on a smartphone.*

**Table:** 

Answers	Number of respondents	Proportion of respondents (%)
1: Strongly disagree 2: Disagree 3: Agree 4: Strongly agree 88: Do not know 99: No answer	476 316 457 710 41 520	24.3 16.1 23.3 36.2 - -

*T39: I do more tasks on a desktop computer or laptop rather than on a smartphone.*

**Table:** 

Answers	Number of respondents	Proportion of respondents (%)
1: Strongly disagree 2: Disagree 3: Agree 4: Strongly agree 88: Do not know 99: No answer	512 319 457 671 41 520	26.1 16.3 23.3 24.3 - -

*T40: Do you know about and use the following smart solutions: apps to manage utility bills,* e.g. *to read meters*

**Table:** 

Answers	Number of respondents	Proportion of respondents (%)
1: Do not know what apps are 2: Know about them but have never used them 3: Have used them at least once 4: Have used them several times 99: No answer	597 525 25 112 1261	47.4 41.7 2.0 8.9 -

*T41: Do you know about and use the following smart solutions: health monitoring apps,* e.g. *to monitor sleep*

**Table:** 

Answers	Number of respondents	Proportion of respondents (%)
1: Do not know what health monitoring apps are 2: Know about them but have never used them 3: Have used them at least once 4: Have used them several times 99: No answer	555 625 32 47 1261	44.1 49.6 2.5 3.7 -

*T42: Do you know about and use the following smart solutions: receive emergency alerts, make emergency calls*

**Table:** 

Answers	Number of respondents	Proportion of respondents (%)
1: Do not know about smart alerts 2: Know about them but have never used them 3: Have used them at least once 4: Have used them several times 99: No answer	593 636 21 9 1261	47.1 50.5 1.7 0.7 -

*T43: Do you know about and use the following smart solutions: to make complaints in public spaces,* e.g. *to report potholes*

**Table:** 

Answers	Number of respondents	Proportion of respondents (%)
1: Do not know about such smart solutions 2: Know about them but have never used them 3: Have used them at least once 4: Have used them several times 99: No answer	834 391 24 10 1261	66.2 31.1 1.9 0.8 -

*T44: Do you use online banking?*

**Table:** 

Answers	Number of respondents	Proportion of respondents (%)
0: No 1: Yes 2: Do not know what online banking is	1546 941 33	61.3 37.3 1.3

*T45: Do you use a digital bank?*

**Table:** 

Answers	Number of respondents	Proportion of respondents (%)
0: No 1: Yes 2: Do not know what a digital bank is	2201 82 237	87.3 3.3 9.4

*T46: Do you know about and use the following smart solutions while travelling: online maps*

**Table:** 

Answers	Number of respondents	Proportion of respondents (%)
1: Do not know about online maps 2: Know but them but have never used them 3: Have used them at least once 4: Have used them several times 99: No answer	294 355 94 516 1261	23.3 28.2 7.5 41.0 -

*T47: Do you know about and use the following smart solutions while travelling: route planner*

**Table:** 

Answers	Number of respondents	Proportion of respondents (%)
1: Do not know about route planners 2: Know about them but have never used them 3: Have used them at least once 4: Have used them several times 99: No answer	258 335 100 566 1261	20.5 26.6 7.9 45.0 -

*T48: Do you know about and use the following smart solutions while travelling: electronic tickets on public transport*

**Table:** 

Answers	Number of respondents	Proportion of respondents (%)
1: Do not know about electronic tickets 2: Know about them but have never used them 3: Have used them at least once 4: Have used them several times 99: No answer	419 616 74 150 1261	33.3 48.9 5.9 11.9 -

*T49: Do you know about and use the following smart solutions while travelling: carpooling services*

**Table:** 

Answers	Number of respondents	Proportion of respondents (%)
1: Do not know about carpooling services 2: Know about them but have never used them 3: Have used them at least once 4: Have used them several times 99: No answer	404 757 58 40 1261	32.1 60.1 4.6 3.2 -

*T50: Do you know about and use the following smart solutions while travelling: shared transport (scooters, bikes)*

**Table:** 

Answers	Number of respondents	Proportion of respondents (%)
1: Do not know about shared transport 2: Know about this but have never used it 3: Have used this at least once 4: Have used this several times 99: No answer	501 716 26 16 1261	39.8 56.9 2.0 1.3 -

*T51: Do you know about and use the following smart solutions while travelling: purchase of digital parking tickets*

**Table:** 

Answers	Number of respondents	Proportion of respondents (%)
1: Do not know about digital parking tickets 2: Know about them but have never used them 3: Have used them at least once 4: Have used them several times 99: No answer	425 566 70 198 1261	33.8 45.0 5.6 15.7 -

### 2.3 Administration

*A1: To what extent do you agree with the following statement regarding public administration: I prefer to do day-to-day tasks in an office rather than online.*

**Table:** 

Answers	Number of respondents	Proportion of respondents (%)
1: Not at all 2: Mostly disagree 3: Mostly agree 4: Totally agree 88: Do not know 99: No answer	323 478 393 794 12 520	16.2 24.0 19.8 40.0 - -

*A2: To what extent do you agree with the following statement regarding public administration: I prefer to do my day-to-day tasks online rather than over the phone.*

**Table:** 

Answers	Number of respondents	Proportion of respondents (%)
1: Not at all 2: Mostly disagree 3: Mostly agree 4: Totally agree 88: Do not know 99: No answer	518 426 493 542 21 520	26.8 21.5 24.9 27.4 - -

*A3: To what extent do you agree with the following statement regarding public administration: I prefer to do my day-to-day tasks in an office rather than over the phone.*

**Table:** 

Answers	Number of respondents	Proportion of respondents (%)
1: Not at all 2: Mostly disagree 3: Mostly agree 4: Totally agree 88: Do not know	280 482 564 1151 43	11.3 19.5 22.8 46.4 -

*A4: Summary variable of people's preferred means of public administration clustered by LCA using variables A1–3.*

**Table:** 

Answers	Number of respondents	Proportion of respondents (%)
1: 1st Online; 2nd Over the phone; 3rd In an office 2: 1st Online; 2nd In an office; 3rd Over the phone 3: 1st In an office; Joint 2nd Over the phone & Online 4: 1st In an office; 2nd Online; 3rd Over the phone 5: 1st In an office (almost everyone); 2nd Over the phone; 3rd Online (almost nobody) 99: No answer	334 399 368 270 600 549	16.9 20.2 18.7 13.7 30.4 -

The variable A4 summarizes the variables A1–3 in the same way as D19, T5 and T33 do using LCA. Variable A4 makes a distinction between respondents whose preferred means of public administration differ. The three variables measure the preferred means of public administration by a pairwise comparison, which can be used to rank the three means. The categories of this variable show the differences between the rankings of the three means. A group of respondents (334 people in category 1) prefer to do day-to-day tasks online rather than over the phone and least of all in person in an office.

*A5: To what extent do you agree with the following statement regarding public administration: If possible, I avoid contacting the customer service team.*

**Table:** 

Answers	Number of respondents	Proportion of respondents (%)
1: Not at all 2: Mostly disagree 3: Mostly agree 4: Totally agree 88: Do not know	766 654 618 452 30	30.8 26.3 24.8 18.1 -

*A6: To what extent do you agree with the following statement regarding public administration: If possible, I avoid handling matters over the phone.*

**Table:** 

Answers	Number of respondents	Proportion of respondents (%)
1: Not at all 2: Mostly disagree 3: Mostly agree 4: Totally agree 88: Do not know	361 663 630 834 32	14.5 26.6 25.3 33.5 -

*A7: To what extent do you agree with the following statement regarding public administration: If possible, I avoid handling matters by post.*

**Table:** 

Answers	Number of respondents	Proportion of respondents (%)
1: Not at all 2: Mostly disagree 3: Mostly agree 4: Totally agree 88: Do not know	641 729 659 463 28	25.7 29.3 26.4 18.6 -

*A8: To what extent do you agree with the following statement regarding public administration: If possible, I avoid handling matters online.*

**Table:** 

Answers	Number of respondents	Proportion of respondents (%)
1: Not at all 2: Mostly disagree 3: Mostly agree 4: Totally agree 88: Do not know 99: No answer	521 472 425 574 8 520	26.2 23.7 21.3 28.8 - -

*A9: I consider it extremely important to be able to choose the means of public administration that is the most convenient for me.*

**Table:** 

Answers	Number of respondents	Proportion of respondents (%)
1: Not at all 2: Not really 3: Usually 4: Always 88: Do not know	145 212 698 1419 46	5.9 8.6 28.2 57.3 -

*A10: Do you know a phone number to call to handle your public administration?*

**Table:** 

Answers	Number of respondents	Proportion of respondents (%)
0: No 1: Yes 88: Do not know	2101 398 21	84.1 15.9 -

*A11: Have you ever handled your public administration over the phone?* (If A10=1)

**Table:** 

Answers	Number of respondents	Proportion of respondents (%)
0: Never 1: Only once 2: Several times 88: Do not know 99: No answer	111 148 137 2 2122	28.0 37.4 34.6 - -

*A12: Do you know a web page to handle your public administration?*

**Table:** 

Answers	Number of respondents	Proportion of respondents (%)
0: No 1: Yes 88: Do not know 99: No answer	1265 701 34 520	64.3 35.7 - -

*A13: Have you ever used a webpage to handle your public administration?* (If A12=1)

**Table:** 

Answers	Number of respondents	Proportion of respondents (%)
0: Never 1: Only once 2: Several times 88: Do not know 99: No answer	134 85 480 1 1820	19.2 12.2 68.6 - -

*A14: Do you have access to a customer portal to handle your public administration?*

**Table:** 

Answers	Number of respondents	Proportion of respondents (%)
0: No 1: Yes 88: Do not know 99: No answer	1532 978 7 3	61.0 39.0 - -

*A15: Which of the following statements is characteristic of your access to a customer portal to handle public administration? (If A14 = 1)*

**Table:** 

Answers	Number of respondents	Proportion of respondents (%)
1: I only use one when necessary 2: I use one and so do others on my behalf 3: Only others use one on my behalf 4: I never use one 99: No answer	800 91 29 58 1542	81.8 9.3 3.0 5.9 -

*A16: Private access to a customer portal to handle public administration.*

**Table:** 

Answers	Number of respondents	Proportion of respondents (%)
0: No 1: If A10 = 1 and A11 = 1	1720 800	68.3 31.7

*Would achieving the following improvement objectives help you to manage your public administrative affairs more efficiently? (multiple-choice question)*

**Table:** 

Subquestion	0: No	1: Yes	99: No answer
A17: Shorter waiting times in offices A18: Shorter travel times A19: Possibility to deal with cases outside working hours A20: Reduction in the number of documents A21: Reduction in administrative time A22: Reduction in procedural costs A23: Increase in the number of possibilities for managing administrative tasks over the phone A24: Increase in the number of mobile phone applications (apps) A25: Increase in the possibility of online administration A26: Possibility of personal contact with an administrator A27: Making the administrative process clearer A28: Simplifying the filling in of forms A29: Making forms easier to read A30: Accessibility to an office A31: Provision of a play area in the customer lounge A32: Proactive services (e.g. email notifications before documents expire)	739 1097 1110 807 896 870 1152 1195 1111 1028 916 797 1036 1206 1230 1176	522 164 151 454 365 391 109 66 150 233 345 464 225 55 31 85	1259 1259 1259 1259 1259 1259 1259 1259 1259 1259 1259 1259 1259 1259 1259 1259

*Which two development goals are most important to you? (ranking-type question)*

**Table:** 

Subquestion	0: Not ranked	1: Rank 1	2: Rank 2	99: No answer
A33: Shorter waiting times in offices A34: Shorter travel times A35: Possibility to deal with cases outside working hours A36: Reduction in the number of documents A37: Reduction in administrative time A38: Reduction in procedural costs A39: Increase in the number of possibilities for managing administrative tasks over the phone A40: Increase in the number of mobile phone applications (apps) A41: Increase in the possibility of online administration A42: Possibility of personal contact with an administrator A43: Making the administrative process clearer A44: Simplifying the filling in of forms A45: Making forms easier to read A46: Accessibility to an office A47: Provision of a play area in the customer lounge A48: Proactive services (e.g. email notifications before documents expire)	800 1057 1050 858 929 885 1095 1112 1053 1026 954 841 1078 1121 1131 1107	239 33 58 146 100 144 20 11 47 71 99 131 12 4 1 12	99 48 30 134 109 109 23 15 38 41 85 166 48 13 6 19	1382 1382 1382 1382 1382 1382 1382 1382 1382 1382 1382 1382 1382 1382 1382 1382

*A49: To what extent would you like to try the following improvement with regard to managing your public administrative tasks: using an online chatbot to get information*

**Table:** 

Answers	Number of respondents	Proportion of respondents (%)
1: I would never try it 2: I would only try it if it would benefit me (e.g. save time) 3: I would like to try it 88: Do not know 99: No answer	339 350 233 88 1510	36.7 38.0 25.3 - -

*A50: To what extent would you like to try the following improvement with regard to managing your public administrative tasks: online administration via a video call with an administrator*

**Table:** 

Answers	Number of respondents	Proportion of respondents (%)
1: I would never try it 2: I would only try it if it would benefit me (e.g. save time) 3: I would like to try it 88: Do not know 99: No answer	303 381 276 50 1510	31.6 39.7 28.7 - -

*A51: To what extent would you like to try the following improvement with regard to managing your public administrative tasks: use of an administration terminal at a customer service desk instead of face to face with an administrator*

**Table:** 

Answers	Number of respondents	Proportion of respondents (%)
1: I would never try it 2: I would only try it if it would benefit me (e.g. save time) 3: I would like to try it 88: Do not know 99: No answer	351 352 240 67 1510	37.2 37.3 25.5 - -

*A52: To what extent would you like to try the following improvement with regard to managing your public administrative tasks: via a video call with an administrator at a customer service desk*

**Table:** 

Answers	Number of respondents	Proportion of respondents (%)
1: I would never try it 2: I would only try it if it would benefit me (e.g. save time) 3: I would like to try it 88: Do not know 99: No answer	356 321 281 52 1510	37.2 33.5 29.3 - -

*A53: How important is the following to you in terms of public administration: to book an appointment*

**Table:** 

Answers		Number of respondents	Proportion of respondents (%)
1: Not important 2: Not very important 3: Quite important 4: Very important 88: Do not know		286 543 785 870 36	11.5 21.9 31.6 35.0 -

*A54: How important is the following to you in terms of public administration: to get help from an office when filling in forms and completing paperwork*

**Table:** 

Answers	Number of respondents	Proportion of respondents (%)
1: Not important 2: Not very important 3: Quite important 4: Very important 88: Do not know	200 394 861 1037 28	8.0 15.8 34.6 41.6 -

*A55: How important is the following to you in terms of public administration: to receive an e-mail or text message when a document is about to expire*

**Table:** 

Answers	Number of respondents	Proportion of respondents (%)
1: Not important 2: Not very important 3: Quite important 4: Very important 88: Do not know 99: No answer	149 285 740 815 11 520	7.5 14.3 37.2 41.0 - -

*A56: How important is the following to you in terms of public administration: to receive an e-mail or text message when the document you have requested is ready*

**Table:** 

Answers	Number of respondents	Proportion of respondents (%)
1: Not important 2: Not very important 3: Quite important 4: Very important 88: Do not know	317 310 796 1065 32	12.7 12.5 32.0 42.8 -

*A57: How important is the following to you in terms of public administration: to be able to pay by credit card at a customer service desk*

**Table:** 

Answers	Number of respondents	Proportion of respondents (%)
1: Not important 2: Not very important 3: Quite important 4: Very important 88: Do not know	531 398 748 812 31	21.3 16.0 30.1 32.6 -

*A58: How important is the following to you in terms of public administration: to be able to pay online*

**Table:** 

Answers	Number of respondents	Proportion of respondents (%)
1: Not important 2: Not very important 3: Quite important 4: Very important 88: Do not know 99: No answer	545 459 546 435 15 520	27.5 23.1 27.5 21.9 - -

*A59: How important is the following to you in terms of public administration: to receive a confirmation e-mail when a document has been sent electronically*

**Table:** 

Answers	Number of respondents	Proportion of respondents (%)
1: Not important 2: Not very important 3: Quite important 4: Very important 88: Do not know 99: No answer	278 242 663 788 29 520	14.1 12.3 33.6 40.0 - -

*A60: How important is the following to you in terms of public administration: to be able to save all electronic documents on your own computer*

**Table:** 

Answers	Number of respondents	Proportion of respondents (%)
1: Not important 2: Not very important 3: Quite important 4: Very important 88: Do not know 99: No answer	333 309 656 676 26 520	16.9 15.7 33.2 34.2 - -

*A61: How important is the following to you in terms of public administration: to be able to have all your documents printed and posted to you*

**Table:** 

Answers	Number of respondents	Proportion of respondents (%)
1: Not important 2: Not very important 3: Quite important 4: Very important 88: Do not know	261 425 673 1132 29	10.5 17.1 27.0 45.4 -

*A62: How important is the following to you in terms of public administration: to be able to track the status of your case online*

**Table:** 

Answers	Number of respondents	Proportion of respondents (%)
1: Not important 2: Not very important 3: Quite important 4: Very important 88: Do not know 99: No answer	346 422 712 506 14 520	17.4 21.2 35.9 25.5 - -

*A63: How important is the following to you in terms of public administration: to communicate with an office in real time via an online interface (*e.g. *chat, Skype, Facebook)*

**Table:** 

Answers	Number of respondents	Proportion of respondents (%)
1: Not important 2: Not very important 3: Quite important 4: Very important 88: Do not know 99: No answer	555 573 529 319 24 520	28.1 29.0 26.8 16.1 - -

*A64: How important is the following to you in terms of public administration: to manage your affairs online from anywhere*

**Table:** 

Answers	Number of respondents	Proportion of respondents (%)
1: Not important 2: Not very important 3: Quite important 4: Very important 88: Do not know 99: No answer	882 613 590 377 38 20	35.8 24.9 24.0 15.3 - -

*A65: Summary variable of the demand for the development of convenience services calculated according to the importance of development and clustered by LCA using variables A53–64.*

**Table:** 

Answers	Number of respondents	Proportion of respondents (%)
1: Not important 2: Quite important 3: Very important 99: No answer	365 852 715 588	18.9 44.1 37.0 -

The variable A65 summarizes the variables A53–64 in the same way as D19, T5, T33 and A4 using LCA. Variable A65 separates respondents that demand developments in administration to different extents. The first group does not consider improvements to be necessary in public administration, the second group expresses a medium level of demand and the third group demands improvements in all the options offered.

## 3 Experimental Design, Materials and Methods

The presented survey was designed to follow the Social Construction of Technology (SCOT) theory highlighting the social aspects and conditions of technological development [3]. The starting point in the experimental design was to go beyond the technological determinism approach based on the decisive role of technology in societal development by considering the social elements and context of technology [4]. The literature on SCOT highlights (1) the importance of the flexible social interpretation of technology by relevant social groups, (2) the influence of social learning on technology-related attitudes and behavioral patterns as well as (3) the key role of the technological frame in encouraging the spread of digital innovation in society [5]. Reflecting on inequality in society, the concept of the digital divide is discussed in detail in the literature with a particular focus on connectivity, accessibility, literacies, content, networks and communication [6,7]. Openness to technological systems is an essential influencing factor in the digital transformation of society, as has long been recognized [8].

Nowadays, the digitalization of public administration is taking place worldwide. Members of an innovative society have different attitudes towards new systems. The challenge is that public administration, as a whole, must be accessible to everyone and digitalization must not exclude any marginalized groups of people. The use of e-government developments, stakeholders and potential users should be continuously monitored. An important question is what factors influence the use of e-government systems, that is, what helps and hinders them? Identifying the pull factors will help to describe the potential customers of e-government developments, moreover, clients who cannot be targeted by e-government developments should also be identified.

To the best of our knowledge, by relying on this theoretical basis, no studies have sought and recognized correlations between the proliferation of digital (smart) technologies, changing platforms, channels through which people manage their cases in practice, as well as the relevant demographic and residential factors. The aim of the present survey was to understand the background on the usage of e-government systems in the digital era. In addition, to fill the knowledge gap by applying the overarching measurement methodology, a monitoring system was developed that examines the impacts of e-government-related operational programs supported by the European Union (2014–2020) at the social level. The results of the programs played an important role in implementing the Hungarian Public Administration and Public Service Development Strategy (PAPSDS). The priorities of PAPSDS aim to increase the efficiency of public administration, streamlining its structure and performance as well as reducing administrative burdens, thereby contributing to the creation of a business-friendly economic environment. The program included interventions such as reducing red tape, strengthening e-governance, increasing transparency, reinforcing human resources as well as plans to develop a comprehensive information database for decision-makers in local authorities.

According to the three main elements of SCOT, a dual goal was defined, namely to create groups based on the knowledge and use of smart devices as well as solutions, moreover, to show the relationship between the attributes of respondents (age, place of residence, type of residence, level of education, income, mode of transport, Internet usage and innovation). The questions were formed by considering suggestions from different disciplines, namely technology studies, public administration, management, urban studies and sociology. Data was collected to explore the attitudes of the population towards public administration and the characteristics of public administration (frequency of use, types of channels used, consumer attitudes) based on a nationwide census of the Hungarian adult population.

## Ethics Statements

The interviewers provided information to respondents with regard to the aims of the survey and how the data would be used. Respondents voluntarily and fully anonymously participated in the research. The authors declare that there are no ethical issues with the data presented. The research was carried out following the procedures outlined by the Declaration of Helsinki. The questionnaire design comply with the personal data protection requirements established in the Hungarian Act CXII of 2011 on the right to information self-determination and freedom of information. All research participants worked according to the protocols declared in Code of Ethics of the University of Public Service, Budapest, Hungary. Ethics approval by the institutional committee is not required specifically for this research.

## CRediT authorship contribution statement

**Tamás Kaiser:** Conceptualization, Supervision, Writing – original draft. **László Gadár:** Data curation, Writing – original draft, Visualization, Writing – review & editing.

## Declaration of Competing Interest

The authors declare that they have no known competing financial interests or personal relationships that could have appeared to influence the work reported in this paper.

## Data Availability

Survey data on the attitudes towards digital technologies and the methods of doing citizens’ (governmental) administration tasks (Original data) (Mendeley Data).
